# Association of device measured physical activity with liver fat and stiffness in people with type 1 diabetes

**DOI:** 10.1111/dom.16584

**Published:** 2025-07-14

**Authors:** Ebaa Al Ozairi, Mohammad Irshad, Jumana Alkandari, Etab Taghadom, Anisha Varghese, Dherar Alroudhan, Nasser Alqattan, Stuart R. Gray

**Affiliations:** ^1^ DAFNE & Clinical Care Research & Trials Unit Dasman Diabetes Institute Kuwait City Kuwait; ^2^ Amiri Hospital Ministry of Health Kuwait City Kuwait; ^3^ School of Cardiovascular and Metabolic Health University of Glasgow Glasgow UK

**Keywords:** liver fat fraction, liver stiffness, physical activity, type 1 diabetes

## Abstract

**Aims:**

This study aimed to investigate the association between physical activity (PA) metrics and liver fat and stiffness in people with type 1 diabetes (T1D).

**Materials and Methods:**

People with T1D attending clinics or participating in ongoing research at the Dasman Diabetes Institute in Kuwait were invited to participate. Liver fat fraction and stiffness were assessed using magnetic resonance imaging and elastography, respectively, liver enzymes were quantified and PA metrics were assessed over 7 days with a wrist‐worn accelerometer. Associations of PA with liver fat, stiffness and enzyme biomarkers were examined using multiple linear regression.

**Results:**

A total of 173 participants with T1D (mean age: 40.3(14.3) years, mean body mass index (BMI): 28.8(4.8) kg/m^2^, mean HbA1c: 7.7(1.3) %, male: 52%) were included. After adjusting for age, sex and BMI, overall PA was negatively associated with liver fat fraction (B = −0.09, *p* = 0.04), liver stiffness (B = −0.02, *p* < 0.001), AST (B = −0.23, *p* = 0.03) and GGT (B = −0.50, *p* = 0.03). The intensity gradient (B = −0.40, *p* = 0.01) and light PA (B = −0.001, *p* = 0.04) were negatively associated with liver stiffness. Moderate to vigorous physical activity (MVPA) was negatively associated with liver stiffness (B = −0.003, *p* = 0.001), AST (B = −0.06, *p* = 0.01) and GGT (B = −0.10, *p* = 0.02). After mutual adjustment, overall PA remained associated with liver fat (B = −0.13, *p* = 0.01), stiffness (B = −0.01, *p* = 0.01) and ALT (B = −0.38, *p* = 0.04), but no associations remained with the intensity gradient.

**Conclusions:**

Promoting PA, particularly MVPA, in T1D may help with the maintenance of liver health.

## 1 INTRODUCTION

Metabolic dysfunction‐associated steatotic liver disease (MASLD), previously known as non‐alcoholic fatty liver disease (NAFLD), is the most common liver disease worldwide, affecting more than 25% of the global population.^1^, ^2^ Although MASLD is primarily seen in people with obesity and type 2 diabetes (T2D),^1^ in recent decades MASLD has also been rising among people with type 1 diabetes (T1D). Indeed, it has been reported that the prevalence of MASLD in T1D ranges from 2.1% to 18.6%, when diagnosed by magnetic resonance imaging (MRI), and from 19% to 31%, when diagnosed by ultrasonography.^2^, ^3^

Hepatic lipid accumulation occurs in MASLD when the rate of hepatic lipogenesis, driven by increased uptake of free fatty acids (FFAs) and enhanced triglyceride synthesis, exceeds the capacity for triglyceride oxidation or efflux as very‐low‐density lipoproteins (VLDLs).^4^ In T1D, this process occurs due to several mechanisms, including hepatic and peripheral insulin resistance, hyperglycaemia‐induced regulation of lipid metabolism, altered hepatic insulin delivery and lipoprotein abnormalities.^2^, ^5^, ^6^ Moreover, frequent episodes of non‐severe hypoglycaemia in T1D are associated with subsequent weight gain^7^, ^8^ and fluctuations in blood glucose levels contribute to metabolic imbalances, which may promote the accumulation of fat in the liver, thereby increasing the risk of MASLD.^9^ This ectopic fat accumulation triggers inflammatory responses and oxidative stress within the liver, which can lead to severe liver disease, such as non‐alcoholic steatohepatitis (NASH), fibrosis, cirrhosis and an increased risk of liver failure or hepatocellular carcinoma.^10^ In addition, people with T1D with MASLD are at higher risk of cardiovascular disease (CVD) and chronic kidney disease (CKD).^11^, ^12^, ^13^ Therefore, strategies to mitigate hepatic lipid accumulation and prevent the progression of fatty liver disease in people with T1D are of the utmost importance.

Once such strategy is physical activity (PA), which plays a pivotal role in diabetes management and the prevention of diabetes related comorbidities.^14^ PA and the distribution of time spent at different intensities of PA, the so called intensity gradient, have been shown to be associated with better glycaemic control and glycaemic variability in people with T1D.^15^, ^16^ Regular aerobic exercise has been shown to significantly reduce liver fat in people without diabetes,^17^, ^18^ and similar results have been observed in people with T2D.^19^, ^20^, ^21^ However, there is comparatively little data in people with T1D. A recent study, in people with T1D, found no association between overall PA and MASLD,^22^ although participating in sports was associated with a lower degree of insulin resistance and lower odds for risk of MASLD.^22^ In this study PA was quantified by a questionnaire, with no studies, to our knowledge, where PA was quantified by devices such as accelerometers. Therefore, the current study aims to (1) compare accelerometer measured PA levels between people with T1D with and without MASLD or metabolic dysfunction‐associated fibrosis (MAF) and (2) explore the association between PA metrics and liver fat and stiffness in people with T1D.

## 2 METHODS

### 2.1 Study setting and participants

People with T1D (aged ≥18 years), attending clinics or participating in ongoing research at the Dasman Diabetes Institute, Kuwait, between 2023 and 2024, were invited to participate in this study. People with a history of alcohol consumption or viral hepatitis or any other factors which can influence liver disease were excluded from the study.

As noted in the ADA position statement^14^ blood glucose responses to PA and exercise are highly variable and to maintain glycaemic balance during and after PA frequent blood glucose monitoring, alongside pre‐activity checks of blood glucose and ketone levels, are recommended. The current study quantifies habitual PA levels, but does not involve a structured exercise intervention, but to ensure participant safety all were enrolled in the Dose Adjustment for Normal Eating (DAFNE) programme, a structured education programme for people with T1D. As part of the programme, participants received comprehensive training on glucose and ketone monitoring. The DAFNE team maintained regular contact with participants, reviewed insulin therapy, monitored adverse events and ensured adherence to safety protocols, including pre‐exercise glucose and ketone testing when indicated.

During a study visit, demographic and clinical data were collected, and participants were provided with an accelerometer to wear for seven consecutive days. The study's purpose and procedures were explained thoroughly, both verbally and in writing, and all participants provided written informed consent. Ethical approval for the study was obtained from the Dasman Diabetes Institute Ethical Review Committee, and the study adhered to the principles outlined in the Declaration of Helsinki.

### 2.2 Demographic and biomedical data

Demographic and biomedical data were obtained from the participants' electronic health records. Age was calculated from the date of birth, and body mass index (BMI, kg/m^2^) was calculated using measured body mass and height. Clinical data, including HbA1c, alanine aminotransferase (ALT), aspartate aminotransferase (AST), γ‐glutamyltransferase (GGT), alkaline phosphatase (ALP), estimated glomerular filtration rate (eGFR), creatinine, FIB‐4 index, total cholesterol, high‐density lipoprotein (HDL) cholesterol, LDL cholesterol and triglycerides, were collected.

### 2.3 Imaging techniques

MRI was performed at the Dasman Diabetes Institute using a Signa Artist (GE Healthcare, USA) system. Trained and experienced radiologists, who were blinded to other study data, conducted all MRI examinations. Participants were instructed to fast for at least 4 h prior to the MRI to reduce potential physiological variability. A torso phased‐array coil was positioned over the abdomen as participants lay supine during imaging. Two MRI techniques were employed: chemical‐shift‐encoded MRI (Double Dixon technique) to estimate hepatic proton‐density fat fraction (PDFF or FF) for diagnosing metabolic‐associated fatty liver disease (MAFLD), and magnetic resonance elastography (MRE) to assess liver fibrosis and stiffness. Significant MAF was defined as MRE stiffness of ≥2.97 kilopascal (kPa),^23^, ^24^ while MAFLD was defined as a fat content (FC) of ≥5%.^25^

### 2.4 Accelerometery

Participants were provided with a GENEActiv Original accelerometer and instructed to wear it continuously (24 h/day) for a period of 7 days. The accelerometer was set to record at a frequency of 100 Hz. Data processing was performed using the GGIR package to generate summary metrics for PA.^26^ Acceleration data collected was calibrated to local gravity using the methods established by van Hees et al.^27^ PA levels were quantified using previously described methods^28^, ^29^ with the intensity gradient calculated according to published methods.^30^ A valid day was defined as having more than 16 h of data, and participants with fewer than three valid days or with missing data for any 15‐min period of the 24‐h cycle were excluded. These inclusion criteria have previously been shown to ensure the validity of the data to represent habitual PA levels.^31^

Overall PA (total volume of PA) was quantified using the acceleration throughout the day, calculated as the Euclidean Norm Minus One (ENMO) in milli gravitational units (m*g*), thus simply reflecting all movement throughout the day. The intensity gradient is a measure of how much time people spend at different intensities of PA. A negative intensity gradient reflects more time performing lower intensity activity compared with high intensity, with a less negative gradient reflecting a more even distribution of times spent at the different intensities. In the general population, people spend more time performing lower intensity activity and therefore have a more negative intensity gradient.^30^

### 2.5 Statistical analysis

Normality of the data was assessed using the Shapiro–Wilk test. PA metrics were compared between groups with or without MAFLD/MAF using the Mann–Whitney U test and effect sizes reported as Cohen's d. Multiple linear regression analysis was used to assess associations of PA metrics with liver fat, liver stiffness and liver enzyme markers, with two models: unadjusted (model 1), adjusted for age, sex and BMI (model 2) and adjusted for model 2 + intensity gradient (when acceleration was exposure) or acceleration (when intensity gradient was exposure) (model 3). Statistical significance was set at *p* < 0.05. All statistical analyses were performed using R and SPSS software.

## 3 RESULTS

This study included 173 people with T1D, with 90 males and 83 females. The clinical and demographic characteristics of the participants are presented in Table 1. Participants' ages ranged from 20 to 74 years, with a mean age of 40.3 ± 14.3 years. The duration of diabetes ranged from 2 to 51 years, with a mean duration of 18.2 ± 9.2 years. HbA1c ranged from 4.8 to 12.3% with the mean 7.7 ± 1.3%, and BMI ranged from 18.2 to 46.3 kg/m^2^ with mean of 28.8 ± 4.8 kg/m^2^. The overall PA was 24.5 ± 8.0 m*g*, which included time spent in light PA of 232.2 ± 98.4 min/day and moderate to vigorous physical activity (MVPA) of 66.6 ± 43.1 min/day. The mean intensity gradient was −2.3 ± 0.3. The mean liver stiffness was 2.3 ± 0.5 kPa, while the liver fat fraction was 4.1% ± 4.7%. Some differences were noted between males and females, with systolic blood pressure, creatinine, triglycerides, ALT, GGT, liver stiffness and inactivity higher in males and eGFR, HDL cholesterol and AST/ALT ratio higher in females.

**TABLE 1:** Baseline demographics total and by sex.

	Total (*n* = 173)	Male (*n* = 90)	Female (*n* = 83)	*p*‐value
	Mean (SD)	Mean (SD)	Mean (SD)	
Age (years)	40.3 (14.3)	40.6 (14.3)	39.8 (14.3)	0.718
Diabetes duration (years)	18.2 (9.2)	18.4 (8.5)	17.9 (10.1)	0.766
Height (cm)	165.8 (9.3)	171.9 (7.3)	159.2 (6.4)	<0.001
Weight (kg)	79.3 (16.0)	85.9 (16.3)	72.1 (12.1)	<0.001
BMI (kg/m^2^)	28.8 (4.8)	29.0 (4.9)	28.5 (4.7)	0.449
Systolic BP (mmHg)	123.9 (12.7)	125.8 (12.0)	121.8 (13.3)	0.040
Diastolic BP (mmHg)	74.5 (8.1)	75.5 (8.0)	73.3 (8.1)	0.085
HbA1C (%)	7.7 (1.3)	7.7 (1.1)	7.8 (1.4)	0.723
eGFR (mL/min/1.73 m^2^)	105.5 (24.3)	100.1 (25.3)	111.3 (21.7)	0.002
Creatinine (μmol/L)	73.9 (43.6)	88.6 (53.7)	57.7 (18.5)	<0.001
Total cholesterol (mmol/L)	4.4 (1.0)	4.3 (1.1)	4.5 (0.9)	0.098
Triglycerides (mmol/L)	1.0 (0.7)	1.1 (0.8)	0.8 (0.4)	0.001
HDL cholesterol (mmol/L)	1.5 (0.4)	1.3 (0.4)	1.7 (0.4)	<0.001
LDL cholesterol (mmol/L)	2.4 (0.9)	2.4 (1.1)	2.4 (0.8)	0.870
AST (u/L)	21.0 (11.3)	22.2 (12.4)	19.7 (9.9)	0.140
ALT (u/L)	29.7 (16.6)	33.4 (17.1)	25.7 (15.2)	0.002
AST/ALT ratio	0.8 (0.3)	0.7 (0.3)	0.8 (0.3)	0.003
FIB4 index	0.6 (0.4)	0.6 (0.4)	0.6 (0.4)	0.353
ALP (u/L)	80.2 (24.2)	81.6 (24.0)	78.7 (24.4)	0.434
GGT(u/L)	30.4 (25.7)	36.1 (30.3)	24.1 (17.6)	0.002
Fat fraction (%)	4.1 (4.7)	4.6 (5.4)	3.5 (3.8)	0.155
Livers stiffness (kPa)	2.3 (0.5)	2.4 (0.6)	2.1 (0.4)	0.009
Overall physical activity (m*g*)	24.5 (8.0)	24.7 (8.1)	24.3 (7.9)	0.749
Light physical activity (LPA, (min/day)	232.2 (98.4)	221.0 (96.4)	244.4 (99.8)	0.119
Moderate to vigorous physical activity (MVPA, min/day)	66.6 (43.1)	72.2 (44.0)	60.6 (41.5)	0.077
Intensity Gradient	−2.3 (0.3)	−2.2 (0.3)	−2.3 (0.3)	0.754
Inactivity (min/day)	739.7 (158.7)	768.1 (160.0)	708.8 (152.3)	0.014

MASLD was present in 17.9% of participants while MAF was present in 8.1%. No cases of severe MAF were observed. People with MASLD/MAF had significantly lower overall PA (mean differences (MD) = −5.2 mg, 95% confidence interval (CI) −8.0, −2.4, *p* = 0.001), MVPA (MD = −29.8 min /day, 95% CI −44.7, −15.0, *p* < 0.001) and light PA (MD = −43.5 min /day, 95% CI −78.3, −8.6, *p* = 0.048), with no differences in intensity gradient (Table 2). In males with MASLD and/or MAF, overall PA (*p* < 0.001), MVPA (*p* < 0.001) and light PA (*p* = 0.02) were significantly lower, while inactivity (*p* < 0.001) was significantly higher compared to males without MASLD and/or MAF. No differences were noted in females (Supplementary Table S1).

**TABLE 2:** Distribution of PA metrics in people with T1D with and without MAF and MASLD.

	MASLD and MAF (*n* = 39)	No MAF or MSLD (*n* = 134)	Mean Difference	95% CI of Difference		Cohen's d^a^	*p* ^b^
	Mean (SD)	Mean (SD)		Lower	Upper		
Overall physical activity (mg)	20.5 (6.2)	25.7 (8.1)	−5.2	−8.0	−2.4	0.67	0.001
Light physical activity (min/day)	198.5 (80.8)	242.0 (101.2)	−43.5	−78.3	−8.6	0.45	0.048
Moderate to vigorous physical activity (min/day)	43.5 (30.8)	73.4 (43.9)	−29.8	−44.7	−15.0	0.72	<0.001
Intensity Gradient	−2.3 (0.3)	−2.2 (0.3)	−0.05	−0.15	0.05	0.18	0.277
Inactivity (min/day)	821.2 (171.2	715.9 (147.2)	105.3	50.4	160.2	0.69	<0.001

Abbreviations: MAF, metabolic dysfunction‐associated fibrosis; MASLD, metabolically dysfunction‐associated steatotic liver disease.

^a^ Effect size.

^b^ Mann–Whitney U test.

The associations between PA metrics and liver fat fraction, liver stiffness and liver enzymes levels are presented in Table 3. In model 1 overall PA was negatively associated with liver fat fraction (B = −0.11, 95% CI = −0.19 to −0.02, *p* = 0.02), liver stiffness (B = −0.02, 95% CI = −0.03 to −0.01, *p* < 0.001), AST (B = −0.22, 95% CI = −0.43 to −0.01, *p* = 0.04) and GGT (B = −0.57, 95% CI = −1.1 to −0.09, *p* = 0.02) but not ALT, FIB4, ALP. After adjustment for age, sex and BMI, overall PA remained associated with liver fat fraction (B = −0.09, 95% CI = −0.17 to −0.003, *p* = 0.04), liver stiffness (B = −0.02, 95% CI = −0.03 to −0.01, *p* < 0.001), AST (B = −0.23, 95% CI = −0.45 to −0.02, *p* = 0.03) and GGT (B = −0.50, 95% CI = −1.0 to −0.07, *p* = 0.03) (model 2). The intensity gradient was negatively associated with only liver stiffness (B = −0.39, 95% CI = −0.67 to −0.10, *p* = 0.01), and this association remained after adjustment (B = −0.40, 95% CI = −0.67 to −0.12, *p* = 0.01) in model 2. After mutual adjustment with intensity gradient in model 3, overall PA remained associated with fat fraction (B = −0.13, 95% CI = −0.23 to −0.03, *p* = 0.01), liver stiffness (B = −0.01, 95% CI = −0.03 to −0.003, *p* = 0.01) and ALT (B = −0.38, 95% CI = −0.74 to −0.02, *p* = 0.04) but the association of intensity gradient with liver stiffness was no longer present.

**TABLE 3:** Associations of physical activity metrics with the liver fat fraction (%), stiffness (pKa) and liver enzymes biomarkers.

	Model 1			Model 2			Model 3		
	95.0% CI for B			95.0% CI for B			95.0% CI for B		
	B	LB, UB	*p*	B	LB, UB	*p*	B	LB, UB	*p*
Fat fraction (%)									
Overall PA (mg)	−0.11	−0.19, −0.02	0.02	−0.09	−0.17, −0.003	0.04	−0.13	−0.23, −0.03	0.01
LPA (min/day)	−0.01	−0.01, 0.001	0.07	−0.004	−0.01, 0.003	0.24			
MVPA (min/day)	−0.02	−0.03, −0.001	0.04	−0.01	−0.03, 0.002	0.08			
IG	0.03	−2.5, 2.6	0.98	0.24	−2.2, 2.6	0.84	2.2	−0.58, 5.0	0.12
Inactivity (min/day)	0.01	0.003, 0.01	0.001	0.01	0.002, 0.01	0.005			
Liver stiffness (pKa)									
Overall PA (mg)	−0.02	−0.03, −0.01	<0.001	−0.02	−0.03, −0.01	<0.001	−0.01	−0.03, −0.003	0.01
LPA (min/day)	−0.001	−0.002, −0.0003	0.01	−0.001	−0.002, −0.00004	0.04			
MVPA (min/day)	−0.003	−0.005, −0.001	0.002	−0.003	−0.005, −0.001	0.001			
IG	−0.39	−0.67, −0.10	0.01	−0.40	−0.67, −0.12	0.01	−0.16	−0.49, 0.17	0.33
Inactivity (min/day)	0.001	0.0001, 0.001	0.02	0.0004	−0.0001, 0.001	0.09			
AST (u/L)									
Overall PA (mg)	−0.22	−0.43, −0.01	0.04	−0.23	−0.45, −0.02	0.03	−0.24	−0.49, 0.01	0.06
LPA (min/day)	−0.003	−0.02, 0.01	0.71	−0.002	−0.02, 0.02	0.82			
MVPA (min/day)	−0.05	−0.09, −0.01	0.02	−0.06	−0.10, −0.02	0.01			
IG	−3.2	−9.3, 2.9	0.30	−3.3	−9.4, 2.7	0.28	0.36	−6.8, 7.6	0.92
Inactivity (min/day)	0.004	−0.01, 0.02	0.48	0.003	−0.01, 0.01	0.64			
ALT (u/L)									
Overall PA (mg)	−0.28	−0.59, 0.03	0.08	−0.26	−0.56, 0.04	0.09	−0.38	−0.74, −0.02	0.04
LPA (min/day)	−0.02	−0.04, 0.01	0.23	−0.01	−0.03, 0.02	0.53			
MVPA (min/day)	−0.05	−0.11, 0.01	0.08	−0.06	−0.12, −0.002	0.04			
IG	0.26	−8.7, 9.2	0.95	0.32	−8.3, 9.0	0.94	6.2	−4.0, 16.4	0.23
Inactivity (min/day)	0.01	−0.01, 0.02	0.25	0.003	−0.01, 0.02	0.71			
FIB4‐ index									
Overall PA (mg)	−0.006	−0.013, 0.001	0.08	−0.004	−0.01, 0.001	0.14	−0.002	−0.01, 0.003	0.40
LPA (min/day)	−0.0004	−0.001, 0.0002	0.21	−0.00004	−0.0004, 0.0004	0.84			
MVPA (min/day)	−0.001	−0.003, −0.0002	0.02	−0.001	−0.002, 0.0002	0.11			
IG	−0.13	−0.32, 0.06	0.18	−0.10	−0.23, 0.04	0.15	−0.06	−0.22, 0.10	0.45
Inactivity (min/day)	0.0003	−0.0001, 0.001	0.15	0.00001	−0.0002, 0.0003	0.98			
GGT (u/L)									
Overall PA (mg)	−0.57	−1.0, −0.09	0.02	−0.5	−1.0, −0.07	0.03	−0.53	−1.1, 0.02	0.06
LPA (min/day)	−0.02	−0.06, 0.02	0.39	−0.005	−0.04, 0.03	0.81			
MVPA (min/day)	−0.10	−0.19, −0.01	0.03	−0.10	−0.19, −0.02	0.02			
IG	−8.9	−22.7, 5.0	0.21	−8.2	−21.5, 5.1	0.23	0.20	−15.6, 16.0	0.98
Inactivity (min/day)	0.03	0.01, 0.06	0.013	0.02	−0.002, 0.05	0.08			
ALP (u/L)									
Overall PA (mg)	−0.25	−0.71, 0.21	0.28	−0.24	−0.69, 0.21	0.30	−0.12	−0.66, 0.41	0.65
LPA (min/day)	−0.01	−0.04, 0.03	0.73	−0.01	−0.04, 0.03	0.79			
MVPA (min/day)	−0.05	−0.13, 0.04	0.30	−0.06	−0.14, 0.03	0.19			
IG	−7.5	−20.6, 5.5	0.26	−7.8	−20.5, 4.9	0.23	−5.9	−21.1, 9.4	0.45
Inactivity (min/day)	0.01	−0.02, 0.03	0.59	0.01	−0.02, 0.03	0.62			

*Note*: Model 1: unadjusted. Model 2: adjusted for sex, age and BMI, Model 3: Model 2 plus Overall PA and IG mutually adjusted.

Abbreviations: ALP, alkaline phosphatase; ALT, alanine transaminase; AST, aspartate transaminase; BMI, body mass index; FF, fat fraction; FIB‐4 index, fibrosis‐4 index; GGT, gamma‐glutamyl transferase; IG, intensity gradients; LPA, light physical activity; MVPA, moderate to vigorous physical activity; Overall PA, overall physical activity.

Light PA was negatively associated with liver stiffness (B = −0.001, 95% CI = −0.002 to −0.0003, *p* = 0.01) in model 1, and this association remained after adjustment (B = −0.001, 95% CI = −0.002 to −0.0004, *p* = 0.04) in model 2. No other associations were seen for light PA. In model 1 MVPA was negatively associated with liver fat (B = −0.02, 95% CI = −0.03 to −0.001, *p* = 0.04), liver stiffness (B = −0.003, 95% CI = −0.005 to −0.001, *p* = 0.002), AST (B = −0.05, 95% CI = −0.09 to −0.01, *p* = 0.02), FIB‐4 (B = −0.001, 95% CI = −0.003 to −0.0002, *p* = 0.02) and GGT (B = −0.10, 95% CI = −0.19 to −0.01, *p* = 0.03), with no associations with ALP, ALT. After adjustment for age, sex and BMI (model 2), MVPA remained associated with liver stiffness (B = −0.003, 95% CI = −0.005 to −0.001, *p* = 0.001), AST (B = −0.06, 95% CI = −0.10 to −0.02, *p* = 0.01), GGT(B = −0.10, 95% CI = −0.19 to −0.02, *p* = 0.02) and, while no association was seen in model 1, ALT (B = −0.06, 95% CI = −0.12 to −0.002, *p* = 0.04), with no other associations seen.

Inactivity was positively associated with liver fat fraction (B = 0.01, 95% CI = 0.003 to 0.01, *p* = 0.001), liver stiffness (B = 0.001, 95% CI = 0.0001 to 0.001, *p* = 0.02) and GGT (B = 0.03, 95% CI = 0.01 to 0.06, *p* = 0.01) in model 1, and the association with liver fat fraction (B = 0.01, 95% CI = 0.002 to 0.01, *p* = 0.005), remained after adjustment, but no other associations were present in model 2. No other associations were seen for inactivity.

## 4 DISCUSSION

The current study provides valuable insights into the relationship between PA and liver health in people with T1D. The findings reveal that overall PA, including MVPA and light PA, was significantly lower in people with T1D and MASLD/MAF. On top of this, the findings showed associations between various accelerometry‐derived PA metrics and biomarkers of liver health, detailing how different aspects of PA are related to components of liver health. The study emphasizes the importance of promoting PA, particularly MVPA and reducing inactivity to mitigate the risk of liver‐related complications in people with T1D.

As mentioned, this study has objectively measured PA and there are no similar studies available in people T1D to compare with the present findings. There are a few studies in people without diabetes that focused on the objective measures of PA and liver health, specially MASLD and fatty liver index.^32^, ^33^ A population‐based cohort study compared accelerometer‐derived PA levels in people with MASLD, assessed by the fatty liver index scores and reported that those diagnosed with MASLD had significantly lower levels of overall PA, including MVPA.^32^ Another study used the UK biobank data and reported accelerometer‐derived MVPA was significantly lower in people with NAFLD.^33^ Overall, therefore, it appears clear people with MASLD have lower levels of PA, which we have now demonstrated in people with T1D, and as PA is important for liver health this may be associated with poorer markers of liver health.

Indeed in the present study, the negative associations observed of overall PA with both liver fat fraction and liver stiffness support the suggestion that engagement in PA may play a protective role in liver health. Notably, these association persisted even after adjusting for key confounders such as age, sex and BMI. Moreover, after mutual adjustment for intensity gradient (model 3), overall PA remained significantly associated with liver fat fraction and liver stiffness, reinforcing the notion that engaging in PA, regardless of the distribution of intensity, is beneficial for reducing liver fat and stiffness. Although intensity gradient was initially associated with liver stiffness, this association was no longer present after adjusting for overall PA (model 3), showing that this initial association was not independent of overall PA levels. These findings were reflected in broadly similar associations in MVPA, but not always light PA, which indicates that the intensity, but not the distribution of this intensity which was also not different in people with MASLD/MAF, at which PA is performed may be important for its benefits to liver health.

The current findings are supported by self‐reported PA data where people with T1D participating in sports had a lower risk of MASLD, although associations with general PA were not seen.^22^ Another study reported that the people with diabetes (both T1D and T2D) who had low self‐reported PA were more likely to have MASLD.^34^ To our knowledge, this is the first study in T1D that reported the association of device‐measured overall PA and its intensity levels with liver fat and stiffness. These findings are supported by a study conducted in people with obesity, where they observed that MVPA, but not light PA, was associated with lower total body fat, visceral fat and liver fat.^18^ In people with T2D, some head‐to‐head randomized control trials (RCTs) have shown that both aerobic and resistance training interventions significantly reduce hepatic fat content and increase insulin sensitivity.^19^, ^20^ Similarly, Bacchi et al. reported that both aerobic and resistance exercise training equally reduced liver fat in people with T2D, and this reduction was accompanied by mild but significant improvements in both anthropometric and metabolic features.^19^ On top of this, a 10 milligravity per hour higher average acceleration, a measure of overall PA, equivalent to 2500 additional steps per day, was associated with a 44% reduction in liver disease progression and a 69% reduction in progression to cirrhosis in people without diabetes.^35^ Similarly, another study based on the UK Biobank reported that each 30‐min per day increase in MVPA was associated with 38% lower odds of hepatic steatosis (OR = 0.62).^36^

The current study also explored the association between PA metrics and liver enzyme biomarkers, finding negative associations of overall PA and MVPA with AST and GGT levels. As with liver fat and stiffness, no associations were seen with light PA, again indicating an intensity dependent effect of PA on the liver. The similarity of these findings with liver fat and stiffness is perhaps not surprising, as we have previously reported a positive association of ALT, AST and GGT enzymes with elevated liver fat and AST and GGT with liver stiffness in people with T1D.^3^ Elevated AST levels are known independent predictors of hepatic fibrosis and MAFLD in T2D.^37^, ^38^ These findings align with a study in women with T2D, where combined aerobic and resistance exercise was shown to decrease ALT and AST liver enzymes and improve the hepatic steatosis index.^39^ A large cohort study also reported that in people without diabetes, inactive men had higher serum levels of ALT and AST compared to active men.^40^ Additionally, in people without diabetes, moderate‐intensity aerobic exercise training has been shown to significantly reduce AST, ALT and GGT levels.^41^ Studies have demonstrated reductions in AST and ALT levels in people with NAFLD who performed either moderate aerobic exercise, resistance training or combined aerobic exercise training.^42^, ^43^ Thus, the present results, coupled with existing data from other populations, indicate that PA is crucial for maintaining liver health in people with T1D by liver function enzymes and reducing fat accumulation and liver stiffness, but these findings need to be confirmed in an appropriately designed RCT.

The current study also found that a higher time spent sedentary was associated with higher liver fat levels, but not other aspects of liver health. Whilst there are no other studies exploring this relationship in people with T1D, there are similar studies in different populations. For example, in a study of habitually active middle‐aged adults a higher objectively measured sedentary time was associated with a higher liver fat percentage.^44^ On the other hand, in people with overweight/obesity further research has demonstrated that there were no associations between objectively measured sedentary behaviour and liver fat levels.^45^ Similar to the current data, a small study (*n* = 37) found that people with NAFLD were more sedentary than age and sex‐matched healthy control participants.^46^ Alongside the aforementioned MVPA data this would indicate that replacing sedentary behaviours with more MVPA would be an optimal strategy to enhance liver health in people with T1D.

The current study is not able to make recommendations for new thresholds of MVPA for liver health in T1D, beyond the general recommendations to participate in 150 min/week MVPA. However, it is known that increasing MVPA beyond the recommendations results in further health benefits and the current study would support this assertion. At this point, it is worth noting that while increasing MVPA offers valuable health benefits, it is not without risk in people with T1D. It is essential to manage glycaemic variability during and after exercise through appropriate carbohydrate intake, insulin adjustments and regular glucose monitoring before, during and after PA, as recommended in the position statement by the American Diabetes Association (ADA).^14^

The mechanisms by which reducing inactivity and increasing PA reduce hepatic fat and stiffness remain unclear but may involve several biological pathways. A large cohort study in T2D showed that high glycaemic variability increases the risk of MAFLD progression.^47^ Although we did not assess glycaemic variability in this study, our previous research found that people with T1D who spent more time engaged in moderate to higher intensity PA have lower measures of glycaemic variability.^15^ This may be one of the possible reasons for the lower liver stiffness observed in people with higher MVPA levels. Many studies in people with and without T2D report that PA enhances insulin sensitivity in muscle and adipose tissue, which reduces the amount of circulating FFAs reaching the liver^48^ and, thus, by decreasing peripheral insulin resistance, PA prevents excessive fat storage in the liver, thereby reducing intrahepatic triglyceride content.^48^ PA also increases fat oxidation and lowers systemic inflammation.^49^ Increasing fat oxidation reduces the fatty acid influx to the liver, preventing fat accumulation within hepatocytes, with reductions in systemic inflammation improving liver stiffness and overall liver health.^49^ Additionally, exercise enhances mitochondrial function in both skeletal muscle and liver tissue.^50^ Increased mitochondrial enzyme activity (e.g., cytochrome c oxidase, citrate synthase) promotes greater β‐oxidation of fatty acids, reducing the accumulation of toxic by‐products like ceramides and diacylglycerides that promote insulin resistance.^50^ PA also influences liver signalling pathways involved in lipid metabolism, reducing intrahepatic triglycerides and improving serum liver enzymes, particularly in those with fatty liver disease.^51^ In animal models, exercise downregulates key lipogenic pathways such as sterol regulatory element‐binding protein‐1c (SREBP‐1c) and carbohydrate response element‐binding protein (ChREBP), reducing the expression of enzymes like fatty acid synthase (FAS) and acetyl‐coenzyme A carboxylase (ACC),^52^ which are involved in liver fat synthesis. Together, these multiple mechanisms highlight how PA can reduce liver fat accumulation and stiffness, potentially preventing or mitigating the progression of liver diseases in physically active individuals, including those with T1D.

Despite the valuable insights gained from this study, several limitations should be noted. First, the cross‐sectional design prevents establishing causality between PA and liver health outcomes. There is potential for reverse causality within the current analyses as it is possible that poor liver health may result in people being less physically activity, and so caution must be applied in the interpretation of this data. As mentioned previously, longitudinal studies are needed to assess whether changes in PA lead to improvements in liver health over time in people with T1D. Although we adjusted for key confounders such as age, sex and BMI, other factors, including diet, and medication use, were not accounted for, which may also impact liver health outcomes. Moreover, the participants in this study were all Kuwaiti and living in Kuwait and this geographic and ethnic specificity may partially limit the generalizability of the findings. Future research should consider these variables to provide a more comprehensive understanding of the relationship between PA and liver health in T1D. For example, we did not have the statistical power to determine whether associations noted differed by sex, and further work with a larger sample size should investigate this.

Overall, this study highlights the significant association between PA and liver health in people with T1D. These results suggest that promoting PA, especially MVPA, may play a crucial role in reducing the risk of liver‐related complications in people with T1D.

## AUTHOR CONTRIBUTIONS

Design: EA, SGR and MI. Conduct/data collection: MI, JAK, ET, AV, DA, NA and SGR. Analysis: MI and SGR. Writing manuscript: MI, SGR and EA. All authors verified the underlying manuscript data and agreed to publish this manuscript.

## FUNDING INFORMATION

The study was funded by the Kuwait Foundation of Advancement of Science (KFAS) and the Ministry of Health, Kuwait. The funding agency did not influence the study design, data analysis, interpretation or report preparation.

## CONFLICT OF INTEREST STATEMENT

Each author declares that they have no known competing financial interests or personal relationships that could have influenced the work reported in this paper. The authors meet criteria for authorship as recommended by the International Committee of Medical Journal Editors (ICMJE) and did not receive payment related to the development of this manuscript.

## PEER REVIEW

The peer review history for this article is available at https://www.webofscience.com/api/gateway/wos/peer-review/10.1111/dom.16584.

## ETHICS STATEMENT

The study was approved by the Dasman Diabetes Institute Ethical Review Committee, Kuwait (RA HM‐ 2020‐ 018), and followed the guidelines set out in the Declaration of Helsinki. All participants signed the consent and agreed to follow.

## Supporting information


**Table S1.** Distribution of PA metrics in people with T1D with and without MAF and MASLD stratified by sex.

## Data Availability

The data that support the findings of this study are available on request from the corresponding author. The data are not publicly available due to privacy or ethical restrictions.
