# Supplementary material for: Association of device measured physical activity with liver fat and stiffness in people with type 1 diabetes
Source: Diabetes Obes Metab. 2025 Jul 14;27(9):5302–10. doi: 10.1111/dom.16584 (PMC12326898; doi:10.1111/dom.16584)
Supplement: Supplementary file 1 — Table S1. Distribution of PA metrics in people with T1D with and without MAF and MASLD stratified by sex. [file DOM-27-5302-s001.docx]

Supplementary table 1: Distribution of PA metrics in people with T1D with and without MAF and MASLD stratified by sex.

|  | MASLD and MAF | No MAF or MSLD | Mean Difference | 95% CI of Difference | | Cohen's d**†** | P |
| --- | --- | --- | --- | --- | --- | --- | --- |
| Male | n= 23, | n= 67 |  |  |  |  |  |
|  | Mean (SD) | Mean (SD) |  | Lower | Upper |  |  |
| Overall physical activity (mg) | 19.1 (6.4) | 26.6 (7.8) | -7.5 | -11.1 | -4 | -1.01 | <.001 |
| Light physical activity (min/day) | 178.6 (87.7) | 235.5 (95.5) | -57 | -101.9 | -12 | -0.61 | 0.02 |
| Moderate to vigorous physical activity (min/day) | 38.8 (29.7) | 83.6 (42.4) | -44.8 | -63.8 | -25.8 | -1.13 | <.001 |
| Intensity Gradient | -2.3 (0.3) | -2.2 (0.3) | -0.1 | -0.2 | 0 | -0.39 | 0.11 |
| Inactivity (min/day) | 852.3 (180.7) | 739.2 (142.5) | 113.1 | 39.7 | 186.6 | 0.74 | <.001 |
| Female | n= 16 | n= 67 |  |  |  |  |  |
| Overall physical activity (mg) | 22.5 (5.4) | 24.7 (8.4) | -2.2 | -6.6 | 2.2 | -0.28 | 0.43 |
| Light physical activity (min/day) | 227.1 (61.5) | 248.5 (106.9) | -21.3 | -76.7 | 34.1 | -0.21 | 0.77 |
| Moderate to vigorous physical activity (min/day) | 50.3 (31.9) | 63.1 (43.4) | -12.8 | -35.8 | 10.2 | -0.31 | 0.39 |
| Intensity Gradient | -2.2 (0.2) | -2.3 (0.3) | 0.03 | -0.1 | 0.2 | 0.09 | 0.86 |
| Inactivity (min/day) | 776.5 (150.7) | 692.7 (149.3) | 83.8 | 1.0 | 166.6 | 0.56 | 0.12 |

†Effect size

*Mann-Whitney U test

MASLD- metabolically dysfunction associated steatotic liver disease.

MAF- metabolic dysfunction-associated fibrosis
